# Pathways of information transmission among wild songbirds follow experimentally imposed changes in social foraging structure

**DOI:** 10.1098/rsbl.2016.0144

**Published:** 2016-06

**Authors:** Josh A. Firth, Ben C. Sheldon, Damien R. Farine

**Affiliations:** 1 Edward Grey Institute, Department of Zoology, University of Oxford, Oxford OX1 3PS, UK; 2 Department of Collective Behaviour, Max Planck Institute for Ornithology, 78457 Konstanz, Germany; 3 Department of Biology, University of Konstanz, 78457 Konstanz, Germany

**Keywords:** social transmission, information use, social networks, social learning strategies, information spread, mixed species flocks

## Abstract

Animals regularly use information from others to shape their decisions. Yet, determining how changes in social structure affect information flow and social learning strategies has remained challenging. We manipulated the social structure of a large community of wild songbirds by controlling which individuals could feed together at automated feeding stations (selective feeders). We then provided novel ephemeral food patches freely accessible to all birds and recorded the spread of this new information. We demonstrate that the discovery of new food patches followed the experimentally imposed social structure and that birds disproportionately learnt from those whom they could forage with at the selective feeders. The selective feeders reduced the number of conspecific information sources available and birds subsequently increased their use of information provided by heterospecifics. Our study demonstrates that changes to social systems carry over into pathways of information transfer and that individuals learn from tutors that provide relevant information in other contexts.

## 1. Introduction

Many animals use social information when making decisions related to fitness, such as for breeding and foraging [1–5]. Recent studies show that information transmission follows social network structure [2–5]. However, it has remained challenging to assess how changes to social structure directly influence information flow [6] or whether social information use itself—owing to its importance—can drive individuals' social associations [7,8]. If the social information produced by certain associates becomes irrelevant, individuals might modify which of their existing associates they learn from [9] or change associates altogether.

Through experimentally manipulating the social foraging associations occurring among wild songbirds, we test how social information transmission is dependent upon social structure. First, as individuals are predicted to gain new information from those they are in contact with, we test whether birds learnt about new ephemeral food sources predominantly from individuals they were experimentally induced to associate with. Second, while changes in information pathways may reflect changes in contact patterns, this could be exaggerated if individuals adaptably direct their learning towards those that provide relevant information. To test whether birds used this social learning strategy, we quantified whether birds disproportionately acquired information from individuals that could regularly access the same resources as themselves.

## 2. Material and methods

### (a) Study system

The experiment took place in three plots at Wytham Woods, Oxfordshire, UK between September 2013 and March 2014 (see [10] for details). Great tits (*Parus major*), blue tits (*Cyanistes caeruleus*), marsh tits (*Poecile palustris*), coal tits (*Periparus ater*) and Eurasian nuthatches (*Sitta europaea*) were fitted with unique but randomly numbered radio frequent identification (RFID)-transponders when caught during the breeding season or by winter mist-netting [4].

RFID antenna-equipped sunflower seed feeding stations continuously recorded the times of individuals' feeding visits at six locations for 40 days. We then replaced each feeder with two ‘selective’ feeders (placed approx. 100 m apart). Selective feeders had clear flaps locked over the feeding hole, which could be unlocked based on a bird's identity (see the electronic supplementary material, videos). At each site, one selective feeder was programmed to allow access only to birds with even-numbered RFID-tags and the other only allowed odd-numbered birds (but both recorded all visits, irrespective of tag-type). Birds of the same tag-type could access the same feeding stations as one another (termed ‘matched’ dyads), while those of the opposite tag-type could only access different feeding stations from one another (termed ‘mismatched’ dyads). We ran this manipulation for 90 days, during which time birds with odd and even tags remained spatially overlapping but became socially segregated [10], as they quickly learnt which feeders they could access [11].

We constructed one social network from the data before the experimental manipulation (40 days) and one during (90 days). We used machine-learning algorithms to identify distinct flocks visiting each feeder [12,13] and constructed social networks given individuals' co-occurrences in flocks using the simple ratio index [14] defined as 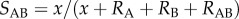, where *S*_AB_ is the social association between birds A and B; *x* is the number of observations of A and B co-occurring together; *R*_A_ and *R*_B_ are the number of times A was recorded without B or B without A and *R*_AB_ is the number of times both birds were simultaneously observed apart.

### (b) Information transfer

To assess information flow [2,5], we used small RFID antenna-equipped feeders to create ephemeral food patches that were freely accessible to all birds and automatically recorded the time when each individual discovered them. Each trial consisted of leaving four such feeders out in random locations (at least 50 m from selective feeders) for four days. We conducted one trial prior to, and four trials during, the manipulation period (leaving 10 or more days between each).

We applied a recent variant [5] of network-based diffusion analysis (NBDA). NBDA estimates the rate, *s*, that individuals learn from knowledgeable tutors (social information transmission) compared to gaining the new information by themselves independently of others (asocial learning [15,16]; see the electronic supplementary material). The multi-network approach [5] estimates different *s* parameters for different types of social associations and hence allows inference about the relative importance of different information transmission pathways.

We first fitted individuals' discovery times of the novel food patches during the pre-manipulation period to the pre-manipulation (baseline) social network. We partitioned the network into four components: (i) matched conspecifics, (ii) matched heterospecifics, (iii) mismatched conspecifics, and (iv) mismatched heterospecifics. We expected to see greater information transfer (i.e. higher *s*) for social associations between conspecifics than between heterospecifics [5], but no difference in *s* values between those of matched and mismatched tag-types (as all individuals could access all pre-manipulation feeding stations).

Next, we fitted patch discovery times during the manipulation period to the pre-manipulation network. Once the manipulation was introduced, individuals become more strongly associated with matched birds (those that could access the same feeding stations as them [10]). Thus, if individuals gain information from those they continually associate with, we expected that mismatched individuals who were previously connected (in the baseline network) would no longer transmit information to one another (lower *s*), and matched individuals would learn from one another faster (higher *s*).

Finally, we fitted patch discovery times to the manipulated network to determine whether individuals also demonstrated a social learning strategy. If the *s* parameter is higher in matched dyads, it suggests that individuals disproportionately copied individuals who would access the same feeding stations as themselves over those they are equally associated with but could only access different feeding stations, i.e. the mismatched individuals they continued to co-occur with during the manipulation (see the electronic supplementary material). This inference is possible because the *s* parameter quantifies the increase in the rate of information transfer *per unit* of social association to knowledgeable individuals [16].

We used an information-theoretic approach when fitting parameters [16], summing Akaike weights to calculate the support for hypotheses regarding information transfer between (i) conspecifics, (ii) heterospecifics, (iii) matched dyads, (iv) mismatched dyads, and combinations of these. Following previous work [2,5], we took a conservative approach to inferring rates of social information use by removing discoveries between individuals that were within 10 min of each other to discard any occasions of birds discovering food together.

## 3. Results

### (a) Data summary

The pre-manipulation social network comprised 10 954 flocking events, with 50 201 co-occurrences among 240 unique individuals. The network during the manipulation comprises 52 483 flocks, with a total of 187 232 co-occurrences among 339 individuals. Novel ephemeral food patches logged 275 discoveries, with 98% of the 148 birds discovering one or more novel patch also occurring at the selective feeders (electronic supplementary material, table S1). Discovering food patches was unbiased relative to tag-type; the distribution of odd-tagged and even-tagged birds among food patches was not significantly different from that expected given the ratio of tag-types (multiple-binomial test *χ*^2^ = 25.11, *p* = 0.07).

### (b) Information transfer

**Figure 1.**
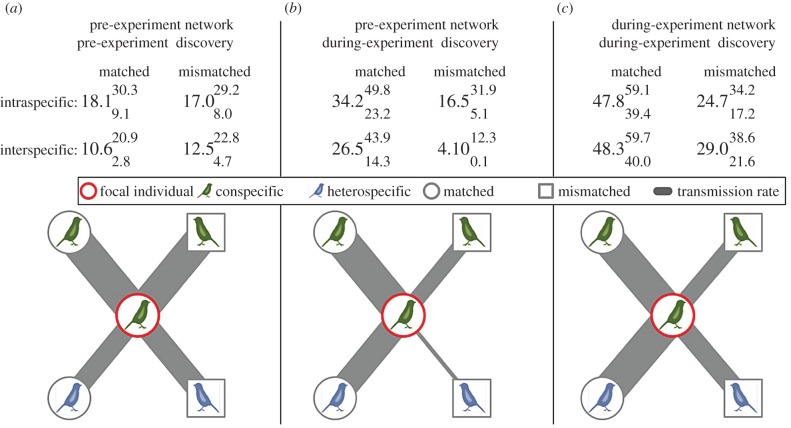
Results of network-based diffusion analysis using: (*a*) pre-experimental network to predict patch discoveries pre-experiment, (*b*) pre-experimental network to predict patch discoveries during experiment and (*c*) network during experiment to predict patch discoveries during the experiment. Grids show model-averaged estimates of *s* for each network type (upper and lower 95% intervals as superscript and subscript, respectively). Diagrams illustrate information transfer between a focal individual (centre) and different types of individual with which they hold equal social associations to within the network (types correspond to grid values). ‘Matched’ refers to individuals who could access the same selective feeder stations as the focal and ‘mismatched’ refers to those who could only access different stations from them. Line thickness shows relative value of *s* within each panel (scaled by maximum *s* estimate); direct comparisons across panels are difficult as parameter estimates also depend on the accuracy and density of the social network.

Before the manipulation began, the rates of information transmission (model-averaged estimates of *s*) following the baseline network were equal between matched and mismatched dyads (figure 1*a*). However, when fitting the baseline network to patch discovery times during the manipulation, pathways of information transmission were notably different (figure 1*b*). While information was still transmitted within the original conspecific and heterospecific networks, including among mismatched dyads, we found strong support for differing rates of information transfer among networks (98% support; see the electronic supplementary material, table S2 for full hypothesis testing). Matched dyads were much more likely to transfer information than mismatched dyads (figure 1*b*). Therefore, individuals acquired information regarding new food sources from those they were experimentally induced to associate with. This effect was particularly evident for heterospecific associations.

Using the experimentally manipulated networks from the selective feeders, we again found support for information transmission across all types of social associations. However, some components of the network were more important for information transfer (electronic supplementary material, table S2). Although *s* values give the rate of information transfer per unit of social association, a significant difference in *s* values between tag-types remained. Information was twice as likely to transfer between matched individuals than between mismatched individuals (figure 1*c*). Yet, while individuals learnt about novel food patches from those that could access the same selective feeding stations as themselves (figure 1*c*), the social associations inferred from flocking co-occurrences at the new food patches after individuals discovered them were equally related to those inferred from the selective feeders for both matched and mismatched dyads (see the electronic supplementary material). Thus, despite the fact that all individuals could access the ephemeral food patches equally, individuals disproportionately learnt from others who could access the same selective feeding sites as themselves, even when controlling for association strength. Additionally, transmission rates within and between species became more similar.

## 4. Discussion

We demonstrate that experimentally manipulating social structure in a wild animal community changes pathways of information transmission. Individuals acquired information about novel food patches from those they were experimentally induced to forage with. Furthermore, over and above the changes to foraging associations, we show preferential use of information provided by birds that produced relevant information in other contexts (i.e. those that could access the same selective feeding stations), despite the fact that the information about freely accessible food was equally valid from all individuals.

A recent laboratory study found that increasing environmental complexity modified social structure, which consequentially increased social transmission [6]. Our study demonstrates that externally driven social segregation linked to an arbitrary phenotype is reflected in patterns of information spread. This could have important implications. For example, cultural divergence can occur when information is transferred primarily within groups [8], which may subsequently restrict gene flow and encourage genetic divergence, thus influencing biological evolution [17].

The types of relationships individuals share are also likely to influence social learning [5]. We found that interspecific social associations that formed before the experiment between birds that subsequently could not feed together (mismatched dyads) were disproportionately less important in predicting information transfer than intraspecific ones (figure 1*b*). This could be owing to some mismatched associations being retained among conspecifics, for example, mated pairs prioritize maintaining their relationship over food access [11]. Nevertheless, birds were more likely to learn from individuals that could access the same feeding stations as themselves than mismatched birds with whom they were equally socially associated (figure 1*c*). This illustrates preferential learning from those that regularly provide relevant information. Individuals also increased their use of information from heterospecifics during the manipulation, potentially to compensate for the decrease in available social information provided by conspecifics (manipulations resulted in a two-thirds decrease in network density). Together, our findings expand the currently limited pool of knowledge regarding how social learning strategies influence information flow in wild populations [4,9].

## Supplementary Material

Supplementary Material

## Data accessibility

Data are available on dryad: http://dx.doi.org/10.5061/dryad.6h4t2.
