# Supplementary material for: Pathways of information transmission among wild songbirds follow experimentally imposed changes in social foraging structure
Source: Biol Lett. 2016 Jun;12(6):20160144. doi: 10.1098/rsbl.2016.0144 (PMC4938043; doi:10.1098/rsbl.2016.0144)
Supplement: Supplementary Material [file rsbl20160144supp1.docx]

**SUPPLEMENTARY INFORMATION: Pathways of information transmission amongst wild songbirds follow experimentally imposed changes in social foraging structure**

Josh A. Firth^1^*, Ben C. Sheldon^1^, Damien R. Farine^1,2,3,^*

^1^Edward Grey Institute, Department of Zoology, University of Oxford, Oxford, OX1 3PS, UK

^2^Department of Collective Behaviour, Max Planck Institute for Ornithology, 78457 Konstanz, Germany

^3^Department of Biology, University of Konstanz, 78457 Konstanz, Germany *Correspondence: joshua.firth@zoo.ox.ac.uk & damien.farine@zoo.ox.ac.uk

**Supplementary Material** - **Supplementary Methods**

**Network-based diffusion analysis**

Network-based diffusion analysis (NBDA) [1, 2] has two key parameters. The first, λ, represents the baseline rate of learning – how fast individuals acquire the trait in the absence of social information. The second, *s*, is the main parameter of interest as it quantifies the increase in the rate of learning for every unit of connection to a knowledgeable tutor. For example, when *s* = 2, an increase of 1 in an individuals social association (or “edge weight”) to knowledgeable individuals means that the individual will learn twice at twice the baseline rate (i.e. twice as fast).

In the multi-network NBDA model, different values of *s* can be inferred for different parts of the network [3]. For example, if a network comprises of two species, then a value of *s* can be inferred for all edges occurring between individuals of the same species, and a value of *s* can be inferred for all edges occurring between individuals from different species. Thus, the same network is now partitioned into two components. If the within-species *s* = 10, then for every increase in edge weight of 1 to knowledgeable conspecifics, an individual is expected to learn at 10 times the baseline rate. If the between-species *s* = 5, then an increase in edge weight of 1 to knowledgeable heterospecifics in the same network will increase the rate of learning by 5 times the baseline rate.

**Inferring experimentally-induced changes in information transfer**

The primary hypothesis we test is whether changing who associates with whom changes the pathways of information transfer. However, there are two potential mechanisms underpinning how pathways of information transfer could change. The first is that the network reconfigures itself because individuals only associate with those they can feed with. If these flocks also search for food together, then information transmission will follow this new pattern of social associations. Previous work suggests that the structure of the social network does change to reflect who can forage with whom [4] (see also next section). Here, we tested if the discovery of novel, freely-accessible, food resources was also more strongly predicted by birds with whom individuals could forage. To do this, we tested which social connections in the pre-manipulated network were inferred to have a higher increase in the rate of learning during the experiment i.e. whether the connections between those who could subsequently access the same feeding stations as one another became more important to information transfer once the experiment began.

Unfortunately, it was not possible to simply test whether the manipulated network was a better predictor of the discoveries than the pre-manipulated network, as other variable may also influence the relationship between an inferred social network and the parameter estimates of information transfer. Notably, the manipulated network was measured temporally closer to the discoveries during the experiment than the pre-manipulated network, and therefore may differ in predictive power for this reason alone. Similarly, the density and accuracy of the inferred social network may also relate to differences in the results of NBDA analysis. Therefore, we base our hypothesis testing exclusively on comparing the relative values of *s* within a network rather than comparing networks or values between different social networks.

**Inferring the presence of a social learning strategy**

An alternative, or potentially contributing, mechanism for observing changes in pathways of information transfer is if individuals pay more attention to those who provide valid information on a regular basis – i.e. if individuals demonstrate a social learning strategy. Note that the two mechanisms are not mutually exclusive, but that this potentially contributing mechanism can be tested independently. As mentioned above, NBDA infers the increase in the rate of information transfer for every unit of connection that an individual has to knowledgeable individuals. Because *s* is a unit-based measure, then differences in rates of learning are estimated independently from differences in the relative strengths of the different social networks. For example, an individual might have a total edge weight of 3 to knowledgeable tutors with the same tag, and a total edge weight of 1 to knowledgeable tutors with an opposing tag type. Given the first mechanism (previous paragraph), we would expect that this individual should learn 3 times faster from matched individuals than mismatched individuals. If that is the case, then the *s* value for these two networks will be the same. By contrast, if the individual learns 9 times faster from matched-access individuals, then *s* for that network will be 3 times higher than *s* for the mismatched dyads’ network. Thus, by applying NBDA to the during-manipulation social network, we can infer how much individuals have changed their behaviour in relation to whom they learn from over and above whom they simply associate with. If we observe similar values of *s* across all networks, then this suggests that the social network manipulation changed patterns of association (individuals associated less with opposing-access individuals), but that individuals did not weigh information from opposing-access associates differently.

**Assessing ability to infer the social learning strategy**

The results of the NBDA analysis, presented in the main body of this paper, used the during-manipulation network to show that individuals were more likely to gain this information from matched associates than mismatched associates. This suggests that birds are more likely to learn from another individual with which they can access the same food on a day-to-day basis in comparison to another individual with which they are equally socially associated to, but that cannot access the same resources as themselves.

However, as with any study of a wild population, the analysis relies on inferred, rather than perfectly known, social associations. Although the robustness of this inference is likely to be relatively high (due to the large amounts of information gathered on the flocking patterns amongst individuals – see *Results*), this may require additional consideration. Specifically, if the inferred social associations between mismatched dyads were underestimated in comparison to the inferred social associations between those of matching dyads, this could confound the conclusions drawn from it.

Therefore, we test if, given the social associations inferred at the selective feeders, considering whether dyads were matched or mismatched would improve the ability to predict social associations in another context. Previous work has shown that the experimental manipulation (selective feeding by tag-type) caused birds to become socially assorted to individuals of the same tag-type (matched) as themselves at the selective feeder sites [4]. It also demonstrated that the social associations at the selective feeders significantly predicted the social associations inferred from the flocking co-occurrences at the food patches (“discovery feeders” – where all individuals could access the food). Consequently, social segregation between those of mismatched tag-types was also present within the social network inferred from the flocks observed at the food patches [4]. Whilst these patterns are known to exist, we had not tested whether social associations inferred from the selective feeders between those matched dyads and mismatched dyads differ in their ability to predict social associations at the discovery feeders.

To do so, we used multiple regression quadratic assignment procedure with double semi partitioning (MRQAP.DSP) [5, 6] which allows a dependent matrix to be regressed over multiple independent matrices, and estimates the effect of each of these. Using this technique, we set the dependent matrix as the social network inferred from the discovery feeders, and the independent matrices as (i) the social network inferred from the selective feeders and (ii) a binary matrix denoting whether dyads were matched or mismatched. We found that, as expected, the selective-feeder social network was a strong predictor of the social associations at the discovery feeders, since the values of the social associations at the selective feeder sites showed a positive relationship with the values inferred from the discovery sites (mrqap.dsp; coeff = 0.394, p<0.001). However, the matrix denoting whether dyads were matched or mismatched showed no significant relationship to the dyadic associations at the discovery sites (mrqap.dsp; coeff = -0.001, p=0.179). Therefore, this illustrates that social associations occurring at the discovery feeders are (i) strongly related to the social associations inferred from the selective feeders, but – when controlling for this – (ii) not related to whether or not individuals could access to same selective feeder sites or not. Hence, accounting for whether or not dyads were matched or mismatched has no significant effect when assessing the relationship between the selective feeder social network and the discovery feeder social network. Therefore, when comparing the social associations inferred from the selective feeders to those in the context of the discovery feeders, social associations between mismatching dyads are not significantly underestimated in comparison to those of matched dyads.

**Supplementary Material – Supplementary Tables**

**Table S1.** Details of sample sizes by species for the pre-experimental period, during the experiment, and the total over the entire study period.

|  | No. birds in network at set feeders | | | No. birds discovering ephemeral patches | | | No. of discoveries of ephemeral patches | | |
| --- | --- | --- | --- | --- | --- | --- | --- | --- | --- |
| Species | Pre | During | Total | Pre | During | Total | Pre | During | Total |
| Blue tit | 119 | 178 | 200 | 31 | 64 | 76 | 31 | 109 | 140 |
| Coal tit | 4 | 6 | 7 | 1 | 3 | 3 | 1 | 9 | 10 |
| Great tit | 102 | 136 | 150 | 18 | 67 | 69 | 18 | 127 | 145 |
| Marsh tit | 9 | 12 | 13 | 4 | 7 | 7 | 4 | 15 | 19 |
| Nuthatch | 6 | 7 | 7 | 2 | 7 | 7 | 2 | 15 | 17 |
| Total | 240 | 339 | 377 | 56 | 148 | 162 | 56 | 275 | 331 |

**Table S2.** Model support (weights) for various hypotheses of social transmission when using i) The pre-experimental network to predict pre-experimental patch discoveries (‘pre-pre’), ii) The pre-experimental network to predict patch discoveries during the experiment (‘pre-during’), iii) The during experiment network to predict patch discoveries during the experiment. The support for opposing hypotheses was examined in different tests. A) Support for the various model forms of Non-constant (social transmission) vs. Constant (asocial transmission), and Additive (no interaction between social and asocial acquisition) vs. Multiplicative (interaction present between social and asocial acquisition) (see Hoppitt et al. 2010). B) Support for whether to include each component of the full social network in the model. C) Support for allowing different *s* values (rate of transmission) when considering the associations between matched dyads vs. the associations between mismatched dyads. See Figure 1 for *s* value estimates.

| Test | Hypothesis | i) Pre-Pre | ii) Pre-During | iii) During-During |
| --- | --- | --- | --- | --- |
| A) Model types | Non-constant  Constant | >0.99  <0.01 | >0.99  <0.01 | >0.99  <0.01 |
|  | Additive  Multiplicative | >0.99  <0.01 | >0.99  <0.01 | >0.99  <0.01 |
| B) Networks to include | Intraspecific only  Interspecific only  Both intra & inter | 0.13  0.01  0.87 | <0.01  <0.01  >0.99 | <0.01  <0.01  >0.99 |
|  | Within tag-types only  Between tag-types only  Both within & between tag-types | 0.04  0.10  0.86 | 0.02  <0.01  0.98 | <0.01  <0.01  >0.99 |
| C) S parameters to include | Different for within/between tag-types  Not different for within/between | 0.41  0.59 | 0.98  0.02 | 0.84  0.16 |

**Supplementary Material – Supporting Video Material**

Two compilations are provided. Video 1 shows some examples of when birds use a selective feeder that they are allowed to feed at. In this case, they are of the correct RFID-tag type i.e. they have an odd-numbered RFID tag and the feeder is programmed to allow birds with an odd-numbered RFID tags to feed there. Video 2 shows examples of when birds attempt to access food at a selective feeder they are not allowed to feed at. Here, they are of the wrong RFID-tag type i.e. they have an even-numbered RFID tag and the feeder is programmed to only allow birds with odd-numbered RFID tags to feed there

Descriptions of the video footage at the ‘selective’ feeding stations:

Video 1:

Accessible at link: https://youtu.be/DOXTM4Blfuc

Also provided in Supplementary material as “selective_feeder_access.mp4”

Video showing a selective feeder allowing access to birds. The following process is taking place: i) the bird lands, ii) the RFID antennae reads the unique RFID tag on the birds leg, iii) the RFID tag is recognised as one which is allowed to access the feeder, iv) the solenoid holding the clear flap in front of the feeding hole is activated and the ‘door’ is now unlocked, v) the bird can now push the flap open and access the seed inside, vi) the bird leaves, and the door is relocked as the solenoid is deactivated.

Description of Video 2:

Accessible at link: https://youtu.be/Ldt38f3Q9Ik

Also provided in Supplementary material as “selective_feeder_denied.mp4”

Video showing a selective feeder denying access to birds. The following process is taking place: i) the bird lands, ii) the RFID antennae reads the unique RFID tag on the birds leg, iii) the RFID tag is not recognised as one which is allowed to access the feeder, iv) the solenoid holding the clear flap in front of the feeding hole remains deactivated and the ‘door’ remains locked, v) the bird cannot push the flap open and therefore cannot access the seed inside, vi) the bird leaves, and the door is remains locked as the solenoid is still deactivated.

**Supplementary Information – Supplementary References**

[1] Franz, M. & Nunn, C.L. 2009 Network-based diffusion analysis: a new method for detecting social learning. *P. Roy. Soc. B-Biol. Sci.* **276**, 1829-1836. (doi:10.1098/rspb.2008.1824).

[2] Hoppitt, W., Boogert, N.J. & Laland, K.N. 2010 Detecting social transmission in networks. *J. Theor. Biol.* **263**, 544-555. (doi:10.1016/j.jtbi.2010.01.004).

[3] Farine, D.R., Aplin, L.M., Sheldon, B. & Hoppitt, W. 2015 Interspecific social networks promote information transmission in wild songbirds. *P. Roy. Soc. B-Biol. Sci.* **282**. (doi:10.1098/Rspb.2014.2804).

[4] Firth, J.A. & Sheldon, B.C. 2015 Experimental manipulation of avian social structure reveals segregation is carried over across contexts. *P. Roy. Soc. B-Biol. Sci.* **282**, 20142350. (doi:10.1098/Rspb.2014.2350).

[5] Dekker, D., Krackhardt, D. & Snijders, T.A.B. 2007 Sensitivity of MRQAP tests to collinearity and autocorrelation conditions. *Psychometrika* **72**, 563-581. (doi:10.1007/S11336-007-9016-1).

[6] Farine, D.R. 2013 Animal Social Network Inference and Permutations for Ecologists in R using asnipe. *Methods Ecol. Evol.* (doi:10.1111/2041-210X.12121).
